# Intergroup Contact and Outgroup Humanization: Is the Causal Relationship Uni- or Bidirectional?

**DOI:** 10.1371/journal.pone.0170554

**Published:** 2017-01-24

**Authors:** Dora Capozza, Gian Antonio Di Bernardo, Rossella Falvo

**Affiliations:** 1 Section of Applied Psychology, Department of Philosophy, Sociology, Education, and Applied Psychology, University of Padova, Padova, Italy; 2 Department of Education and Human Sciences, University of Modena and Reggio Emilia, Modena, Italy; Universita Cattolica del Sacro Cuore, ITALY

## Abstract

The attribution of uniquely human characteristics to the outgroup may favor the search for contact with outgroup members and, vice versa, contact experiences may improve humanity attributions to the outgroup. To explore this bidirectional relationship, two studies were performed. In Study 1, humanity perceptions were manipulated using subliminal conditioning. Two experimental conditions were created. In the humanization condition, the unconditioned stimuli (US) were uniquely human words; in the dehumanization condition, the US were non-uniquely human and animal words. In both conditions, conditioned stimuli were typical outgroup faces. An approach/avoidance technique (the manikin task) was used to measure the willingness to have contact with outgroup members. Findings showed that in the humanization condition participants were faster in approaching than in avoiding outgroup members: closeness to the outgroup was preferred to distance. Latencies of approach and avoidance movements were not different in the dehumanization condition. In Study 2, contact was manipulated using the manikin task. One approach (contact) condition and two control conditions were created. The attribution of uniquely human traits to the outgroup was stronger in the contact than in the no-contact conditions. Furthermore, the effect of contact on humanity attributions was mediated by increased trust toward the outgroup. Thus, findings demonstrate the bidirectionality of the relationship between contact and humanity attributions. Practical implications of findings are discussed.

## Introduction

Since the beginning of this century, researchers have intensively investigated humanity attributions to groups, showing that people tend to perceive the outgroups as less human than the ingroups (for reviews, see [1, 2]). This bias, which relates to different types of outgroups (e.g., ethnic and racial outgroups, stigmatized categories, patients in medical contexts) and may occur in the absence of overt conflicts, generally has detrimental consequences, hindering prosocial behaviors and promoting violence and discrimination.

As noted by Haslam and Loughnan [1], researchers have analyzed the types, reasons, and consequences of dehumanization, but little attention has been paid to the issue of how humanity perceptions can be improved. The most extensively investigated intervention strategy has been intergroup contact (for a review, see [3]), revealing a positive relationship between different modes of contact and outgroup humanization. However, the cross-sectional design of most studies performed (e.g., [4, 5]) makes it difficult to establish the direction of causality in the association between contact and humanity perceptions.

In the current work, using experimental designs, we try to show that the relationship between intergroup contact and outgroup humanization is bidirectional, that is: contact improves humanity attributions to the outgroup and favorable humanity attributions promote contact. To our knowledge, this is the first time that the reciprocal relationship between contact and humanity perceptions has been experimentally investigated (for a longitudinal study, see [6]). Our studies may contribute to the research about humanity perceptions by showing that intergroup contact may induce outgroup humanization (regarding this relationship, only two experimental studies have been performed, with different research designs [7, 8], and using children as respondents [8]). Our studies may contribute to the research about intergroup contact by identifying a factor (humanizing perceptions) that promotes intergroup encounters (for other precursors of contact, see [9–12]).

## Infrahumanization and Dehumanization

The flowering of studies and theories exploring humanity perceptions started with Leyens and colleagues’ work [13] on the attribution of uniquely human (secondary) and non-uniquely human (primary) emotions to groups. These authors discovered that group members assign more secondary emotions (e.g., hope, regret) to one’s group than to the outgroup, whereas they do not differentiate the two groups with reference to primary emotions (e.g., excitement, rage). This form of bias has been called infrahumanization (see [2]). The perception that ingroups are characterized by uniquely human features more than outgroups (infrahumanization) applies to emotions, but also traits such as rationality, morality, and conscientiousness (see, e.g., [14]). However, the other groups are not only infrahumanized, they may also be dehumanized, and different forms of dehumanization have been discovered. Outgroups are assimilated to animals when they are perceived as lacking the unique features of the human species (see [15–18]); outgroups are assimilated to machines when they are perceived as lacking the essential features of human nature (e.g., emotionality, vitality, and warmth; see [1, 19], see also [20]). Dementalization may also be revealed, for instance when outgroups are perceived as irrelevant to the fulfillment of one’s goals [21, 22].

Outgroup infrahumanization and dehumanization can strongly damage intergroup relations. It has been found, for instance, that infrahumanization may be associated with aggression, discrimination, and support for violence against the outgroup [23, 24]. In sexual aggressions, the dehumanization of women is associated with men’s proclivity to sexual harassment and rape [25]. In medical contexts, patient infrahumanization may be used by health workers as a strategy to cope with job-related stress [26, 27]. Thus, strategies have to be singled out to curb the humanity bias, which is deeply rooted in the human cognitive system. Initial evidence suggests that humanity perceptions can be improved by making a common ingroup identity salient [4, 28] or weakening the belief that humans are different and superior to animals [29, 30]. Furthermore, an increasing number of studies is showing that outgroup humanization may be achieved by shifting from a social categorization of outgroup members to their individualization, resulting from salience of multiple affiliations (e.g., [31, 32]). However, the most widely investigated strategy has been intergroup contact which, after 60 years of research, has emerged as the most effective psychosocial strategy for ameliorating intergroup relationships (see [33, 34]).

## Intergroup Contact and Humanity Attributions

In this research domain, investigators have analyzed different types of intergroup encounters. The effects of direct contact were studied by Brown and colleagues [6], using a longitudinal research design. These authors discovered that frequent encounters with outgroup members attenuate infrahumanization, measured as the difference between the number of secondary emotions assigned to the ingroup and the outgroup. However, the reverse relationship–from lower infrahumanization to more frequent contact–was not significant. A reliable association between direct contact (quality × quantity) and lower infrahumanization was found by Tam et al. [5] in a survey conducted on Catholics and Protestants in Northern Ireland. Further correlational evidence was provided by Capozza et al.’s [4] studies, in which humanity attributions were measured using uniquely human and non-uniquely human traits.

It is not surprising that both more intimate forms of contact–such as cross-group friendships–and extended contact are related to outgroup humanization [35] (extended contact is the knowledge that ingroup members have close relationships within the outgroup [36]). There is now initial evidence for the effectiveness of imagined contact (see [37]): simply imagining a pleasant encounter with an unknown outgroup member may lead to perceiving the outgroup as more characterized by uniquely human features [7, 8].

Thus, the association between intergroup contact and outgroup humanization has been demonstrated considering different forms of contact, different types of attributes (traits and emotions), and different kinds of outgroups (e.g., ethnic, religious, marginalized groups). But experimental evidence is scarce [7, 8], and it only involves the causal relation from contact to outgroup humanization. In the current studies, we hypothesized that intergroup contact promotes outgroup humanization, and outgroup humanization promotes contact.

We tested these hypotheses by employing experimental procedures, never used in dehumanization and contact research. We used subliminal conditioning to manipulate humanizing perceptions, and a computerized approach training technique to manipulate contact (for approach training techniques, see [38, 39]). We adopted an implicit manipulation of humanity perceptions because we aimed to eschew demand characteristic effects; we adopted an approach training technique to manipulate contact, because we aimed to use similar methods to shape contact/no contact conditions and to measure contact as an outcome of humanity perceptions (contact was measured using an approach/avoidance technique).

Finding a bidirectional relationship between contact and humanity perceptions may have practical implications. Investigators may, in fact, reduce discrimination in society by both favoring contact, which promotes outgroup humanization, and favoring humanizing perceptions, which promote contact.

## The Current Research

To test the hypothesis of a reciprocal relationship between humanity perceptions and intergroup contact, we performed two studies. In Study 1, we manipulated humanity perceptions and observed the effects of this manipulation on contact, measured as approach (vs. avoidance) responses to outgroup members. In Study 2, we manipulated contact by training participants to perform approach movements toward outgroup members (typical outgroup faces, shown on a computer screen) and observed the effects of contact on humanity attributions to the outgroup.

In Study 1, humanity perceptions were manipulated by employing a conditioning procedure (e.g., [40–42]) which involved supraliminal presentations of the conditioned stimuli (CS) followed by subliminal presentations of the unconditioned stimuli (US). As CS, we used typical faces of the outgroup (Moroccans; participants were Italian). The US differed in the humanization and dehumanization conditions. In the humanization condition, the US were words corresponding to uniquely human traits (e.g., rationality, morality), uniquely human emotions (e.g., pride, shame), and concepts unique to the human category (e.g., citizen, humans). In the dehumanization condition, unconditioned stimuli were words corresponding to non-uniquely human traits (e.g., instinct, impulsiveness), non-uniquely human emotions (e.g., pleasure, rage), and concepts unique to animals (e.g., cub, animals). As in evaluative conditioning, the repeated pairing should change the accessibility of automatic associations; in the humanizing condition, repeated pairing should increase the accessibility of outgroup/human concepts associations. We called the dehumanizing condition “dehumanization,” because the US also included uniquely animal concepts.

To assess readiness to contact, we used the manikin task [43]. Participants were instructed to move a little figure (the “virtual self”) on the screen toward or away from outgroup stimuli (typical Moroccan names, e.g., Abdul). Two critical blocks were used. In one block, the participants’ task was to move the manikin, representing the self, toward the outgroup names and away from neutral stimuli (names of geometrical figures); in the other block, their task was to move the manikin away from outgroup names and toward the neutral stimuli. The time between the onset of the stimulus (the outgroup name or the neutral word) and the beginning of the movement was used as the dependent variable: for outgroup names, the shorter this time, the stronger the disposition to approach or avoid outgroup members.

We predicted that, for outgroup stimuli, approach responses would be faster in the humanization than in the dehumanization condition; conversely, avoidance responses should be faster in the dehumanization than in the humanization condition (Hypothesis 1). Furthermore, we predicted that, while in the humanization condition approach responses would be faster than avoidance responses, in the dehumanization condition, avoidance responses would be faster, or the two types of action would have similar latencies (Hypothesis 2). We proposed an alternative for the dehumanization condition because the greater rapidity of avoidance than approach responses could depend on the degree to which the outgroup is recognized as dangerous, a problem not explored in the present study.

To assess the inclination to contact induced by outgroup humanization, we used an implicit rather than explicit measure of approach for two reasons. First, because research has demonstrated that, when evaluative conditioning is used to change rather than form attitudes, it typically impacts implicit, rather than deliberate, responses (see [42, 44]). Second, because implicit assessment is not biased by social desirability concerns. To measure approach/avoidance responses, we used the manikin task because this method is more sensitive and exhibits more criterion validity than other approach/avoidance methods [45], such as the joystick task [46, 47]. Furthermore, in the manikin task, participants have the impression of moving toward other people as it happens when, in real life, they search for contact or are invited to search for contact with outgroup members. As for the outgroup, Moroccans were selected because they represent a large immigration group in Italian society, but also for practical reasons: in constructing the experimental material, in fact, it is easy to find names and faces that are perceived as typically Moroccan.

In Study 2, we simulated contact using the approach training technique (e.g., [38, 48]). With this method participants are trained to approach an outgroup typically by pulling a joystick toward the self when outgroup members are presented. Research has shown that an extensive training generates a more positive attitude toward the outgroup, this effect being mediated by a stronger self-outgroup association [48]. In Study 2, when training participants in approaching the outgroup (Moroccans), we employed the manikin task which, used as a training technique, provides significant results, by shaping positive attitudes [39] and decreasing fear-related responses [49]. We preferred to use the manikin rather than the joystick task because the former offers a simulation of the behaviors featuring real interpersonal encounters.

In Study 2, we created three experimental conditions. In the approach Moroccans (contact) condition, participants were instructed to move the manikin (the self) toward outgroup stimuli (Moroccan faces) and away from neutral stimuli (images of geometrical figures). In the neutral stimuli condition, participants were instructed to move the manikin toward images of furniture and away from geometrical figures. In the sideways control condition, the manikin was moved to the right in response to Moroccan faces and to the left in response to geometrical figures. We added this control condition because any difference between the approach Moroccans condition and the approach furniture condition could depend not on contact experiences, but on the exposure/non-exposure to outgroup cases. In all conditions, the dependent variable was the attribution of uniquely human (e.g., rationality, morality) and non-uniquely human (e.g., instinct, impulsiveness) traits to the outgroup. As demonstrated by pilot studies [4], the two sets of traits we used did not differ in valence.

We hypothesized that the attribution of uniquely human traits to the outgroup would be higher in the contact than the two control conditions, which should not differ from each other (Hypothesis 3). On the basis of previous research [2] showing that infrahumanization does not involve the non-uniquely human dimension, we hypothesized that the attribution of non-uniquely human traits would not differ in the three contact conditions (Hypothesis 4). In testing these hypotheses, we will control for the effects of attitudes, because approach training favors positive outgroup evaluations [38, 48].

A robust effect in contact research is that the relationship between intergroup contact and lower prejudice is mediated by emotional factors, such as reduced anxiety, increased empathy, and increased trust toward the outgroup (see, e.g., [50–52], for direct contact; [8, 53], for imagined contact; [35, 54], for cross-group friendships). In addition, when humanity attributions are the outcome and immigrants are the outgroup, the reliable mediators in the relationship between contact and outgroup humanization turn out to be reduced anxiety [4] and increased trust [8]. Thus, we hypothesized a mediation effect of the two emotions: in the contact condition, the attribution of uniquely human traits to the outgroup should be greater than in the control conditions because contact attenuates anxiety and increases trust toward the outgroup (Hypothesis 5).

One may ask what form of intergroup contact, among those investigated, is reproduced by the manikin’s (the virtual self) approach movement toward the outgroup members (symbolized by typical names in Study 1 and typical faces in Study 2). Clearly, our contact operationalization does not reproduce real direct contact with known or unknown outgroup members. Considering the various forms of indirect contact, the manikin’s movement toward the outgroup does not simulate extended contact (the fact of knowing that ingroup members have friendships within the outgroup [36]); nor does it simulate vicarious contact: the observation of interactions between ingroupers and outgroupers (for a review of these indirect forms of contact, see [55]). The manikin’s movement procedure represents a special form of imagined contact [37, 56]: the participant imagines meeting outgroup members (approach trials). In the approach training in Study 2, participants were also asked to imagine a positive interaction, a task used in the imagined contact research. Thus, our contact measure (Study 1) and contact manipulation (Study 2) reproduce a never used condition of imagined repeated movement toward and imagined repeated interaction with outgroup members. In the two previous studies, adopting an imagined contact manipulation [7, 8], the usual procedure of visualizing a positive interaction with an outgroup member was applied. Interestingly, the approach training technique in Study 2 parallels the no-choice condition in engaging in intergroup contact, that generally induces effects of the same size as those induced by the full-choice and some-choice conditions [34].

## Study 1

In this study, we tested Hypothesis 1 and Hypothesis 2, and investigated whether outgroup humanization promotes contact with outgroup members.

### Method

#### Ethics statement

This study and Study 2 were approved by the Psychological Research Ethics Committee of the University of Padova. All participants gave written informed consent.

#### Participants

Participants were Italian psychology students. Among the students attending a course, 61 (*M*_*age*_ = 22.62, *SD* = 2.17; 41 females and 1 missing answer) volunteered to participate in the experiment. Participants were randomly assigned to either the humanization (*n* = 31) or the dehumanization (*n* = 30) condition.

#### Humanization/dehumanization conditioning

The conditioning paradigm involved supraliminal presentations of the CS followed by subliminal presentations of the US. As CS, we used six typical Moroccan faces (males); as US, we used uniquely human words (humanization condition) or non-uniquely human and animal-related words (dehumanization condition). The uniquely human words were: four uniquely human traits (e.g., rationality, morality; see [4]); six secondary emotions–three positive (e.g., pride, optimism) and three negative (e.g., regret, shame); five words expressing concepts unique to the human species (e.g., humans and citizen [15]). The non-uniquely human or animal-related words were: four non-uniquely human traits (e.g., instinct, impulsiveness [4]); six primary emotions–three positive (e.g., pleasure, excitement) and three negative (e.g., rage, pain); five words expressing concepts unique to the animal category (e.g., animals and cub [15]). In pilot studies, we found that the uniquely human and non-uniquely human traits did not differ in valence [4]; the same result was found for the uniquely human and uniquely animal concepts [15] (the US employed in the humanization and dehumanization conditions are reported in S1 Appendix).

Participants were informed that this first task aimed to test whether the presentation of outgroup faces distracted perceivers’ attention. Participants were then told that they would see Moroccan faces or geometrical figures (ovals) in the center of the computer screen; their task was to indicate whether a target stimulus (a string of Xs) was located above or below the face or figure. On the numeric keypad, participants had to press the 8 (↑) key, when the string was above the face or the figure, and the 2 (↓) key, when it was below. The string was actually a masking stimulus used for the subliminal presentation of the US. Each trial started with a Moroccan face (six faces) or an oval (two ovals) that remained visible until participants responded. Five-hundred ms from the CS appearance, a row of Xs (the pre-mask) was presented (20 ms), immediately replaced by the US (40 ms), that is, a word expressing a uniquely human or non-uniquely human/animal concept, according to the condition. The US was followed by the post-mask: the string of Xs shown for 250 ms. Words used as US and masks were presented parafoveally, that is, they were positioned 2° above or below the fixation point [57] which was the center of the face or the oval. Participants indicated the position of the post-mask by pressing the “above” or “below” key. The inter-trial interval was of 1500 ms. In the critical conditioning trials, the six Moroccan faces were associated with the 15 uniquely human stimuli (humanization condition) or the 15 non-uniquely human stimuli (dehumanization condition). These 90 associations were interspersed with 30 neutral trials (two ovals associated with 15 subliminal words). The experimental trials were preceded by eight practice trials.

#### The manikin task

Participants were told that the second experiment investigated the relationship between Italians and Moroccans. Their task was to move a manikin–a simple drawing of a person–upward or downward on the screen pressing the 8 (↑) or 2 (↓) key. Participants were instructed to imagine being the manikin. At the beginning of each trial, a cross was shown in the center of the screen (500 ms) to center participants’ attention. After the cross presentation, the manikin appeared in either the upper half or the lower half of the screen. Triggered by pressing key 5 on the numeric keypad, a stimulus (a typical Moroccan name or the name of a geometrical figure) appeared in the center of the screen. In one block of trials (approach block), participants were required to move the manikin toward the outgroup stimuli (Moroccan names) and away from the figure names; in another block (avoidance block), they were required to do the opposite: avoid the outgroup stimuli (Moroccan names) and approach the figure names. Participants were instructed to press the relevant key three times: their visual impression was that the manikin was walking toward or away from the target; 500 ms after pressing the key for the third time, all stimuli disappeared from the screen. A 1000 ms inter-trial interval was used (for the procedure used, see [45]).

Both the approach block and the avoidance block included 80 trials. In the approach block, participants approached Moroccan names (40 trials) and avoided geometrical figure names (40 trials): five Moroccan names (e.g., Abdul, Hassan) and five figure names (e.g., square, circle) were used. In half of the 40 trials (either approach or avoidance), when the movement started, the manikin was above the stimulus, in the other half, it was below. For the avoidance block, the approach trials regarded the geometrical figures, while the avoidance trials regarded the outgroup names. The order of presentation of the two blocks was counterbalanced across participants. For each trial, the time between the onset of the stimulus and the first key pressing–which started the manikin’s movement–was calculated. Faster reaction times indicate greater readiness in approaching or avoiding the stimulus.

#### Procedure

Participants, individually examined, were told they would perform two experiments which were combined to make better use of the participant pool: the first experiment focused on attention processes and the second the relationship between Italians and Moroccans. Detailed interviews, conducted upon completing the study, indicated that none of the participants was aware of the relation between conditioning and manikin task. Participants were not aware that words were presented during the subliminal trials, either.

### Results

For the manikin task, latencies from trials with errors or higher than 1500 ms were removed. We submitted latencies to a 2 (condition: humanization vs. dehumanization) × 2 (target: Moroccans vs. geometrical figures) × 2 (action: approach vs. avoidance) ANOVA with repeated measures on the last two factors. The three-way interaction was significant, *F* (1,59) = 4.01, *p* = .05, η^2^_p_ = .06. Therefore, we analyzed findings concerning the two targets separately.

**Fig 1:**
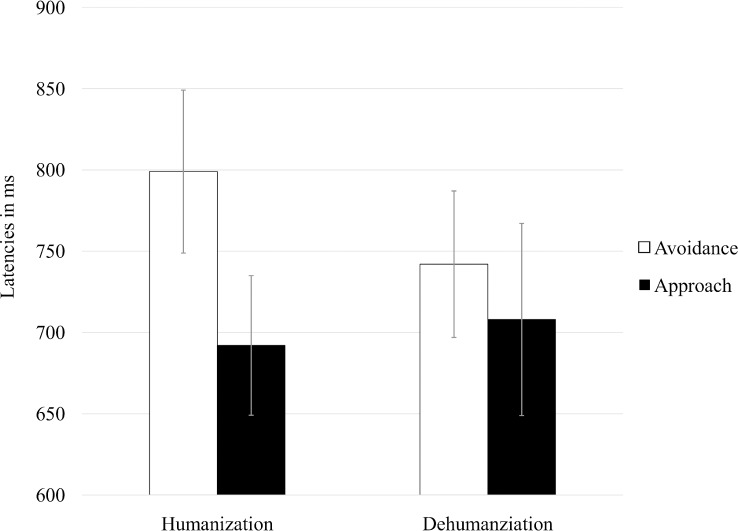
Mean latency scores as a function of the condition (humanization vs. dehumanization) and the type of action (approach vs. avoidance), Study 1. Note: Bars represent 95% confidence intervals.

The main effects and the interaction were nonsignificant when the target was represented by the control stimuli, *F*s < 1. A significant interaction was instead found when the target was the outgroup, *F* (1,59) = 9.09, *p* < .005, η^2^_p_ = .13. Simple effects showed that approach movements toward the outgroup were not faster in the humanization than dehumanization condition, *F* < 1, and avoidance movements were not slower in the humanization condition, *F* (1,59) = 2.81, *p* < .10. However, while in the humanization condition, approach was much faster (*M* = 692 ms, *SD* = 120) than avoidance (*M* = 800 ms, *SD* = 140), *F* (1,59) = 39.30, *p* < .001, η^2^_p_ = .40, in the dehumanization condition, the difference between the two movements did not reach significance, *F* (1,59) = 3.75, *p* < .06, η^2^_p_ = .06 (Fig 1): for approach movements, *M* = 708 ms (*SD* = 162); for avoidance movements, *M* = 742 ms (*SD* = 126). Thus, findings supported Hypothesis 2, but not Hypothesis 1.

#### Supplementary data

Findings showed that the humanizing manipulation mobilized the “desire for contact” with outgroup members: in this condition, in fact, approach movements were faster than separation movements. But, in order to establish that the humanizing manipulation was effective in promoting contact, we need to show that the baseline condition, in our experiment, was that of dehumanization; in other words, we need to show that, independently of any manipulation, participants associated Moroccans with non-uniquely human attributes and animal concepts. Notably, research has documented that repeated category/stereotype pairings do not alter the existing associations, and training conditions, aimed to maintain the stereotypic associations, have the same effects on the outcomes as no-training/control conditions (see [58, 59]; see also [38, 48]).

To control whether Moroccans were associated with uniquely human or non-uniquely human/animal concepts, we gathered supplementary data. Participants were 45 Italian university students (*M*_*age*_ = 22.47, *SD* = 2.67; 24 females); they were required to evaluate the outgroup on four uniquely human traits (morality, rationality, reasoning, intellectual abilities) and four non-uniquely human traits (drive, impetus, impulsiveness, instinct): the same traits used as US in the conditioning procedure (see S1 Appendix). The two sets of traits do not differ in valence, as shown in pilot studies [4]. Participants rated the outgroup on each trait responding on a 7-step scale, for instance, “Moroccans are characterized by morality” (1 = *definitely false*, 7 = *definitely true*; 4 = *neither true nor false*). Alphas were .80 and .68 for uniquely human and non-uniquely human traits, respectively.

We also assessed the automatic associations between Moroccans and human (e.g., human, citizen) and animal (e.g., animal, cub) concepts, by using the Implicit Association Test (IAT; [60]). Implicit techniques allow us to assess the–likely unaware–associations between the outgroup and animal concepts. Participants were instructed to categorize Moroccan and Italian personal names, and human and animal concepts. Five typical Moroccan names and five typical Italian names were used. For the human/animal dimension, we employed five human (bachelor, citizen, human, humans, young boy) and five animal (animal, animals, cub, fauna, specimen) words: the same used as US in the conditioning procedure (see S1 Appendix). In pilot studies [15], we found that the human and animal concepts do not differ in valence. In accordance with IAT procedures, participants were presented with five blocks of practice trials and two blocks of critical trials. The order of critical blocks was counterbalanced across respondents. In one critical block (compatible block), participants were asked to use one key to categorize Moroccan names and animal words and another key to categorize Italian names and human words. In the other critical block (incompatible block), participants were asked to use the same key when categorizing Moroccan names and human words, and another key when categorizing Italian names and animal words. Each critical block consisted of 40 trials. IAT scores were computed according to a scoring algorithm proposed by Greenwald and colleagues [61]. Positive D scores indicate an association between Moroccans and animal concepts.

Regarding traits, means were *M* = 4.02 (*SD* = 1.18), for uniquely human traits, and *M* = 5.02 (*SD* = 0.78), for non-uniquely human traits; only the latter mean was reliably different from the scale mid-point, *t* (44) = 8.78, *p* < .001, *d* = 1.31 (for the uniquely human traits, *t* < 1). Furthermore, the outgroup was perceived as more characterized by non-uniquely human than uniquely human traits, *t* (44) = 4.56, *p* < .001, *d* = 0.68. As to the IAT, the mean of D scores was *M* = 0.53 (*SD* = 0.36), reliably different from zero, *t* (44) = 9.94, *p* < .001, *d* = 1.48. Thus, in the IAT, participants were faster to categorize words in the Moroccan/animal condition than in the Moroccan/human condition.

These findings show that, independently of any manipulation, participants associate the outgroup with non-uniquely human traits and animal concepts. Thus, in Study 1 the dehumanizing condition represents the baseline/control condition, and the different behaviors we found in the humanization condition were an effect of humanizing manipulations.

### Discussion

Study 1 shows that, when the outgroup is associated with uniquely human concepts, movements toward outgroup members are faster than movements away from them (Hypothesis 2), namely, outgroup humanization favors the desire for closeness and contact with outgroup members. However, Hypothesis 1 was not supported; in fact, approach movements were not faster in the humanization than in the dehumanization condition, and avoidance movements were tendentially, but not significantly, faster in the dehumanization condition. We believe that some procedural changes could lead to support Hypothesis 1. For instance, a stronger association between the outgroup and humanity concepts could be obtained if a greater number of outgroup/humanity pairings were used in the conditioning procedure. Kawakami and colleagues [58] employed numerous trials (384) in a training task, aimed to change White participants’ stereotype of Blacks (in the 192 trials concerning Black targets, participants were invited to say “NO,” when the photograph of a Black was paired with a Black stereotype, and to say “YES,” when it was paired with a White stereotype). In other studies, in applying the approach training technique to model new automatic associations between the self and the racial outgroup (White participants), Phills and colleagues [48] used a high number (480) of trials: in half the trials, respondents approached Blacks by pulling a joystick toward the self (when pictures of Blacks were shown); in the other half, they avoided Whites by pushing the joystick away from the self (when pictures of Whites were shown). A similar number of push-responses (about 200) to alcohol pictures was employed by Wiers et al. [62] to shape new tendencies to avoid alcohol among hazardous drinkers (however, Wiers and colleagues [63] obtained a reduction in drinking also using a lower number–about 100 –of push-responses; and see [64] for the attenuation of implicit prejudice against Backs). We believe that a greater number of pairings can create stronger Moroccan/humanity associations, these associations leading to faster approach and slower avoidance responses in the humanizing than in the dehumanizing condition.

Another procedural change could involve the stimuli employed to represent the outgroup. In research aimed to form new outgroup/stereotype or outgroup/evaluation associations, the same type of stimuli (pictures or words) is generally used to portray the outgroup in the intervention and the measure of intervention effects (Black faces were, for instance, employed in [48, 58, 64]). Probably, the outcomes of humanizing manipulation would be stronger if Moroccan faces or Moroccan names were used in both the conditioning procedure and in the approach/avoidance technique.

In Study 1, we have shown that outgroup humanization leads to the desire for contact with outgroup members: in the humanization condition, participants were faster in approaching than in avoiding Moroccan stimuli. We now evaluate whether intergroup contact improves humanity attributions.

## Study 2

In Study 2, we created three experimental conditions: a contact condition in which participants were trained to approach outgroup members, a control condition in which they were trained to approach neutral stimuli, another control condition in which participants performed neutral movements in response to outgroup stimuli. The effects of these manipulations on humanity attributions were measured using uniquely human and non-uniquely human traits.

### Method

#### Participants

Participants were Italian psychology students. Among the students attending a psychology course, 57 (*M*_*age*_ = 25.11, *SD* = 5.01; 42 females) volunteered to participate in the experiment. Each participant was randomly assigned to one of the three conditions: contact condition (*n* = 19), neutral stimuli control condition (*n* = 20), sideways control condition (*n* = 18).

#### The approach training task

For the approach training, the manikin task was used. In the contact condition, participants were instructed to move the manikin (the virtual self) toward Moroccan faces (six faces, the same used in Study 1) and away from geometrical figures (two ovals, the same used in Study 1). In approaching Moroccans, they had to imagine a positive, cooperative interaction. For each face and oval, the manikin was located six times above and six times below the figure. Therefore, participants performed 72 approach movements in response to outgroup stimuli and 24 avoidance movements in response to neutral stimuli. In half of the approach movements and half of the avoidance movements, the manikin was moved upwards, while, in the other half, it was moved downwards. The 96 critical trials were preceded by 12 practice trials (for the procedure of stimuli presentation and manikin’s movement on the screen, see Study 1).

In the control condition (neutral stimuli), participants were instructed to move the manikin toward furniture images (six figures) and away from the two ovals: in 72 trials, participants approached furniture images, in 24, they avoided ovals. In the sideways control condition, participants performed neutral movements both in response to Moroccan faces and in response to the ovals. In 72 trials, they had to move the manikin toward the right in response to Moroccan faces, in 24, they had to move the manikin toward the left in response to the ovals. In half the trials, the manikin was located above the stimulus, while, in the other half, it was located below. Movements were obtained by pressing the 6 (→) or the 4 (←) key on the numeric keypad. The 96 critical trials were preceded by 12 practice trials.

#### Dependent measures

Humanity attributions to Moroccans were measured by using four uniquely human (morality, rationality, reasoning, intellectual abilities) and four non-uniquely human (drive, impetus, impulsiveness, instinct) traits (see Supplementary data in Study 1). Participants rated the outgroup (Moroccans) on each trait by responding on a 7-step scale, for instance: “Moroccans are characterized by rationality” (1 = *definitely false*; 7 = *definitely true*; 4 = *neither true nor false*). Alphas were .76 and .79 for uniquely human and non-uniquely human traits, respectively. For attitudes, participants rated Moroccans on five semantic differential items representing the evaluation factor (good/bad, pleasant/unpleasant, agreeable/disgusting, valuable/worthless, desirable/undesirable). On the 7-step scale, 1 was assigned to the negative and 7 to the positive pole; alpha was .75. To measure intergroup anxiety–one of the two hypothesized mediators–we asked participants how they would feel, if being involved in a social encounter with Moroccans they had never met (see [65]). Participants then indicated the extent to which they would feel anxious or at ease. Six anxiety items (e.g., anxious, uncertain, tense) and six non-anxiety items (e.g., calm, confident, secure; reverse coded) were used (alpha = .88); the 7-step scale was anchored by *not at all* (1) and *very much* (7). For outgroup trust, the second mediator, we used four items (see [35]): “I trust Moroccans,” “I distrust Moroccans” (reverse coded), “I feel I can trust Moroccans,” “Moroccans are unreliable” (reverse coded) (alpha = .84). On the 7-step scale, higher scores express higher levels of outgroup trust. For all the measures used, items were averaged to form reliable composite scores.

#### Procedure

Upon arrival, participants, examined individually, were informed that their first task was to respond to stimuli of different type for an investigation about theories of learning and cognitive processes. Having completed the manikin task, participants were required to answer a questionnaire which measured their perceptions of Moroccans. Upon completing the experiment, participants were probed for insights into the researchers’ hypotheses. None of the participants was aware of the expected effects of training on Moroccan representations.

### Results

**Fig 2:**
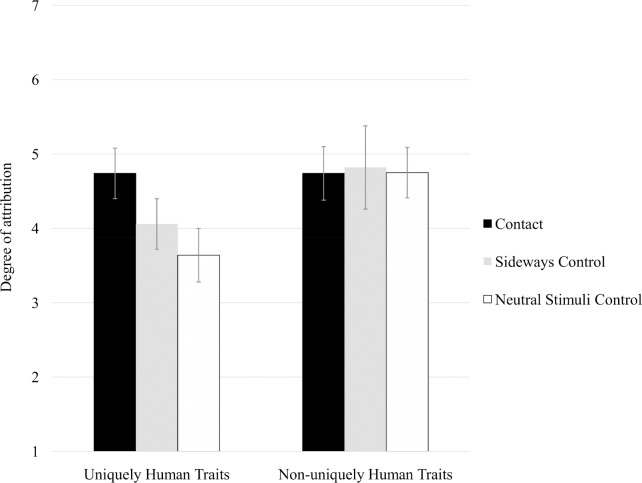
Attribution of uniquely human and non-uniquely human traits to the outgroup as a function of the contact vs. control conditions, Study 2. Note: Bars represent 95% confidence intervals.

To test Hypothesis 3 and Hypothesis 4, a 3 (conditions: contact vs. neutral stimuli control condition vs. sideways control condition) × 2 (traits: uniquely human vs. non-uniquely human) mixed-model ANOVA was applied. The two main effects, *F*s > 4.07, *p*s < .03, η^2^_p_s > .12, were qualified by a significant interaction, *F* (2,54) = 4.15, *p* < .03, η^2^_p_ = .13. Simple effects analysis revealed that the means of the three conditions differed for the uniquely human traits, *F* (2,54) = 10.02, *p* < .001, η^2^_p_ = .27, but not for the non-uniquely humans traits, *F* < 1 (Fig 2). In the case of uniquely human traits, the mean was higher in the contact condition (*M* = 4.74, *SD* = 0.75) than in the sideways control condition (*M* = 4.06, *SD* = 0.74) and neutral stimuli condition (*M* = 3.64, *SD* = 0.82): for the first comparison *t* (35) = 2.78, *p*< .01, *d* = 0.91; for the second, *t* (37) = 4.35, *p* < .001, *d* = 1.39. Means did not differ for the two control conditions, *t* (36) = 1.65, *p* < .11. ANCOVA, applied to the uniquely human traits, using attitudes as the covariate, revealed a significant effect of conditions, *F* (1,53) = 5.89, *p* < .006, η^2^_p_ = .18; findings regarding the comparison between the three conditions replicated those obtained from ANOVA. Thus, Hypotheses 3 and 4 were fully confirmed.

**Fig 3:**
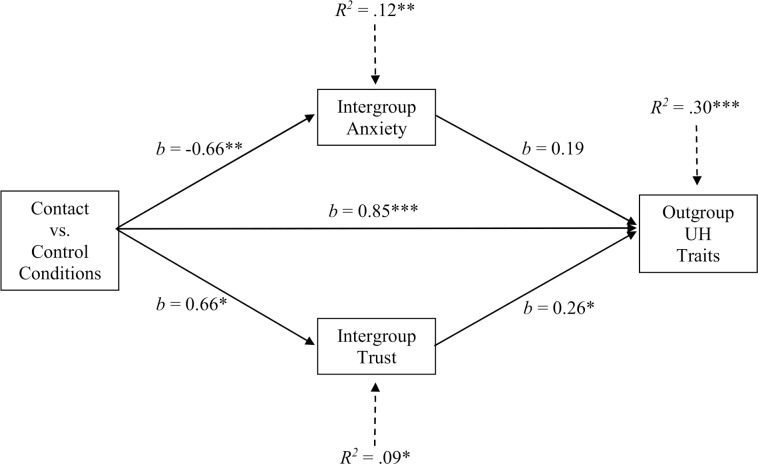
Mediation effects of intergroup emotions in the relationship between contact and the attribution of uniquely human (UH) traits to Moroccans, Study 2. Note: Unstandardized regression coefficients are reported. *** *p* < .001, ** *p* < .01, * *p* < .05.

To test the mediation effects of anxiety and trust toward the outgroup, the macro PROCESS [66] was applied. In the tested model, the independent variable was represented by the experimental conditions (1 was assigned to the contact condition and 0 to the control conditions); the uniquely human traits attributed to Moroccans were the outcome (correlations between the four variables are reported in Table 1). Significance of indirect effects was estimated by using bootstrapping (10,000 resamples) and a 95% bias corrected confidence interval. Findings are shown in Fig 3.

**Table 1:** Zero-order correlations between the variables involved in the mediation model, Study 2.

	1	2	3	4
1. Contact vs. no-contact conditions	-			
2. Anxiety toward the outgroup	-.34**	-		
3. Trust toward the outgroup	.30*	-.61***	-	
4. Uniquely human traits of the outgroup	.48***	-.15	.33*	-

Note: For the contact/no-contact variable, 1 was assigned to the contact condition and 0 to the sideways control condition and the neutral stimuli control condition.

*** *p* < .001

** *p* < .01

* *p* <. 05.

As it appears from the diagram, approach behaviors toward the outgroup (contact condition) both attenuated anxiety and increased the feelings of trust toward Moroccans; trust, in turn, was related to a greater attribution of uniquely human characteristics to Moroccans. The indirect effect of contact via trust was significant; in fact, the 95% confidence interval did not include zero [0.04, 0.38] (the point estimate was 0.18). In contrast, the indirect effect of contact via anxiety was nonsignificant [0.03, -0.43] (the point estimate was -0.12). Thus, Hypothesis 5 was partially supported: the mediation effect of emotions was, in fact, significant for trust but not for anxiety. As to the magnitude of the mediation effect of trust, it was significant when considering two relevant measures of effect size: the partially standardized indirect effect (point estimate = 0.20, 95% confidence interval [0.05, 0.43]), and the ratio of indirect to total effect (point estimate = 0.20 and confidence interval [0.05, 0.50]) (for what effect size indicators to use, when the independent variable is dichotomous and there are multiple mediators, see [66]).

We tested alternative mediation models, in which the attribution of uniquely human traits to Moroccans was the mediator and anxiety or trust were the outcome. In the model on anxiety, the 95% confidence interval included zero [0.36, - 0.25] (point estimate = 0.02) (the indirect path was nonsignificant also when trust was introduced in the model as a covariate). Mediation of humanity attributions was instead significant when the outcome was trust [0.03, 0.59] (point estimate = 0.27). In this alternative model, the path linking humanity attributions to trust was nonsignificant (*b* = 0.30, *p* < .10); however, it became significant (*b* = 0.31, *p* < .04) when anxiety was introduced in the model as a covariate. Thus, findings indicate that anxiety and outgroup humanization were unrelated effects; for trust, both trust explained the relationship between contact and outgroup humanization and outgroup humanization explained the relationship between contact and trust.

### Discussion

In Study 2, we found that repeated approach to outgroup members, while imagining a positive interaction, promoted the attribution of uniquely human traits to the outgroup. This result was also obtained when the effect of outgroup evaluation was controlled. The mere experience of outgroup members (in the sideways control condition) was not sufficient to improve humanity attributions.

The mediation analysis evidenced that the contact manipulation reduced anxiety and increased trust toward the outgroup. This finding, replicating what is generally found in contact research (see, e.g., [50, 52], and [8] for imagined contact), supports the efficacy of our contact manipulation, based on approach behaviors and imagined encounters. However, only outgroup trust was a significant mediator. In the case of anxiety, two antagonistic processes may be at play. On one hand, anxiety could lead to a lower attribution of uniquely human traits, which may justify planned defensive, discriminatory behaviors toward the outgroup, on the other hand, anxiety could lead to a greater attribution of uniquely human traits, which allows the decrease in anxiety (see the nonsignificant path linking anxiety with outgroup humanization in Fig 3). Further studies could test these potential antagonistic effects of anxiety.

In testing the alternative models, we found that as trust mediates the relationship between contact and humanity attributions, so humanity attributions mediate the relationship between contact and trust. In Hypothesis 5, a mediation role of trust was proposed, on the basis of theoretical traditions and findings in the literature on contact (for imagined contact, see, e.g., [8, 53, 67–69]). However, a reciprocal mediation process is possible, a process that can be tested by using longitudinal designs.

## General Discussion

The present studies show that outgroup humanization leads to the desire for contact with outgroup members (in the humanizing condition, approach is preferred to avoidance), and contact with outgroup members leads to outgroup humanization. This is the first time that the bidirectionality of the contact/outgroup humanization connection has been studied using experimental designs (for the only longitudinal study on this relationship, see [6]). Our investigation contributes to the outgroup dehumanization research (for a recent review, see [70]) by experimentally demonstrating that intergroup contact promotes the attribution of uniquely human traits to the outgroup (previous experimental studies regarded children and a different outgroup, see [7, 8]). Our investigation contributes to the intergroup contact research by showing, for the first time, that humanizing perceptions may be an antecedent of the search for contact with outgroup members and, whereas much is known about contact outcomes, knowledge relative to contact precursors is rather limited.

In Study 1, we did not observe that approach behaviors toward the outgroup were faster in the humanization than in the dehumanization condition and avoidance behaviors were faster in the dehumanization condition. We observed that participants liked to be in contact with targets who were good instances of the human category, that is, participants preferred to approach than to avoid them. As mentioned earlier, stronger effects of the humanization manipulation could be obtained if a higher number of outgroup/humanity pairings were used in the conditioning procedure, and the same type of outgroup stimuli (faces or names) were considered in the manipulation and approach/avoidance technique. Future research should replicate Study 1, by performing these procedural changes.

In Study 2, supporting Hypothesis 3, we found that approach movements toward outgroup members, imagining a positive interaction, improved outgroup humanity perceptions. This increase in outgroup humanization, deriving from the manipulation of contact, could actually depend on demand characteristics effects. However, Eyssel and Ribas [71] demonstrated that measures of humanity perceptions (the attribution of secondary or primary emotions), differently from self-report measures of attitudes, are unbiased by demand characteristics and social desirability concerns. In future studies, however, findings could be replicated by using implicit measures of humanity perceptions (see, e.g., [15, 25]).

In Study 2, we also found that outgroup trust, but not intergroup anxiety, played a role in the contact/outgroup humanization relationship: in this relationship, trust was a mediator or, in the alternative model, the outcome of humanizing perceptions evoked by contact. Future longitudinal studies should test the hypothesis that outgroup trust mediates the effects of contact on humanity attributions and humanity attributions mediate the effects of contact on outgroup trust. Regarding the prevalence of trust over anxiety in explaining contact effects (baseline model), this finding is not new: it has been observed, for instance, when attitudes [67, 69] or cooperative intentions [69] were the outcome of contact. For humanity attributions, the prevalence of trust may be explained by the perceptions associated with this emotion: trusting, in fact, implies the recognition that the target is characterized by uniquely human attributes, such as morality and the capacity of understanding other people’s expectations (for outgroup trust, see [72]).

Thus, our studies show that intergroup contact favors outgroup humanization and outgroup humanization favors the desire for contact. This reciprocal causation demonstrates the close link between contact and humanity perceptions. In fact, as it has been insightfully noticed [16], bidirectionality signals that an association is strong and well rehearsed. Another theoretical contribution of the present work involves the antecedents of contact. Research has extensively investigated the effects of contact on prejudice reduction, and the mediators and moderators of these effects (see, e.g., [33, 34]). Less is known about the factors that lead to the search for contact with outgroup members. It has been observed that prior experiences predict later contact (see, e.g., [73]), and opportunities for contact are related to higher levels of meaningful contact (e.g., [74]). A motivational factor, leading people to seek encounters with outgroup members, is the need to achieve learning goals [11]. Our research provides evidence for a further precursor: the perception that the outgroup is characterized by uniquely human features leads to the desire for closeness to its members. We have finally proposed a new form of imagined contact, based on imagination of approach behaviors and positive encounters, a form which is effective in promoting trust and outgroup humanization, and reducing intergroup anxiety.

The practical implications of our findings are evident. If we want ingroup members to be willing to have contact with outgroup members (for instance, Moroccans), in contexts in which contact is likely or required (workplaces, schools), information should be provided about the uniquely human characteristics of the outgroup, for instance, its creativity (the presence of excellent writers in the country), or the outgroup’s country striving to achieve economic growth and social change. These humanity representations should make ingroup members more inclined to search for contact with the outgroup. On the other hand, if we wish to improve humanity attributions, we should promote positive intergroup encounters or imagined simulations of these encounters. Study 2 in this paper, and previous research have demonstrated that contact is effective in favoring outgroup humanizing perceptions.

Despite the encouraging results obtained in our studies, the following limitations should be addressed. First, in the two studies, only one outgroup was considered. Findings should be replicated using other outgroups, for instance, stigmatized groups, such as disabled or homeless people, who are targets of dehumanizing perceptions. Second, we found a short-lived change in humanity perceptions (Study 2). Future research should test the strength of this change by investigating its temporal conditions. Third, in future research, further mediators should be evaluated. Extensive practice in approaching the outgroup could improve humanity perceptions by increasing the association between the self and the outgroup [48] which is similar to the inclusion of the outgroup in the self mechanism: a powerful mediator of contact effects [75]. Despite these limitations, our studies show that both humanity perceptions and intergroup contact are key factors which can effectively ameliorate intergroup relationships in society.

## Supporting Information

S1 Appendix Unconditioned stimuli used in the procedure of subliminal conditioning, Study 1. (DOCX)

S1 Dataset Data from Study 1. (XLSX)

S2 Dataset Data from Study 2. (XLSX)

S3 Dataset Supplementary data. (XLSX)

## References

[1] Haslam N, Loughnan S. Dehumanization and infrahumanization. Annu Rev Psychol. 2014; 65: 399–423. 10.1146/annurev-psych-010213-115045 23808915

[2] Leyens J-P, Demoulin S, Vaes J, Gaunt R, Paladino MP. Infra-humanization: The wall of group differences. Soc Issues Policy Rev. 2007; 1: 139–172.

[3] Capozza D, Falvo R, Di Bernardo GA, Vezzali L, Visintin EP. Intergroup contact as a strategy to improve humanness attributions: A review of studies. TPM Test Psychom Methodol Appl Psychol. 2014; 21: 349–362.

[4] Capozza D, Trifiletti E, Vezzali L, Favara I. Can intergroup contact improve humanity attributions? Inter J Psychol. 2013; 48: 527–541. 10.1080/00207594.2012.688132 22721357

[5] Tam T, Hewstone M, Kenworthy JB, Cairns E, Marinetti C, Geddes L, et al Post-conflict reconciliation: Intergroup forgiveness and implicit biases in Northern Ireland. J Soc Issues. 2008; 64: 303–320.

[6] Brown R, Eller A, Leeds S, Stace K. Intergroup contact and intergroup attitudes: A longitudinal study. Eur J Soc Psychol. 2007; 37: 692–703.

[7] Falvo R, Capozza D, Di Bernardo GA, Pagani A. Can imagined contact favor the “humanization” of the homeless? TPM Test Psychom Methodol Appl Psychol. 2015; 22: 23–30.

[8] Vezzali L, Capozza D, Stathi S, Giovannini D. Increasing outgroup trust, reducing infrahumanization, and enhancing future contact intentions via imagined intergroup contact. J Exp Soc Psychol. 2012; 48: 437–440.

[9] Boccato G, Capozza D, Trifiletti E, Di Bernardo GA. Attachment security and intergroup contact. J Appl Soc Psychol. 2015; 45: 629–647.

[10] Hodson G, Busseri MA. Bright minds and dark attitudes: Lower cognitive ability predicts greater prejudice through right-wing ideology and low intergroup contact. Psychol Sci. 2012; 23:187–195. 10.1177/0956797611421206 22222219

[11] Migacheva K, Tropp LR. Learning orientation as a predictor of positive intergroup contact. Group Process Intergroup Relat. 2013; 16: 426–444.

[12] Turner RN, Dhont K, Hewstone M, Prestwich A, Vonofakou C. The role of personality factors in the reduction of intergroup anxiety and amelioration of outgroup attitudes via intergroup contact. Eur J Pers. 2014; 28: 180–192.

[13] Leyens J-P, Rodriguez AP, Rodriguez RT, Gaunt R, Paladino MP, Vaes J, et al Psychological essentialism and the differential attribution of uniquely human emotions to ingroups and outgroups. Eur J Soc Psychol. 2001; 31: 395–411.

[14] Costello K, Hodson G. Exploring the roots of dehumanization: The role of animal-human similarity in promoting immigrant humanization. Group Process Intergroup Relat. 2010; 13: 3–22.

[15] Capozza D, Andrighetto L, Di Bernardo GA, Falvo R. Does status affect intergroup perceptions of humanity? Group Process Intergroup Relat. 2012; 15: 363–377.

[16] Goff PA, Eberhardt JL, Williams MJ, Jackson MC. Not yet human: Implicit knowledge, historical dehumanization, and contemporary consequences. J Pers Soc Psychol. 2008; 94: 292–306. 10.1037/0022-3514.94.2.292 18211178

[17] Goff PA, Jackson MC, Di Leone BAL, Culotta CM, DiTomasso NA. The essence of innocence: Consequences of dehumanizing Black children. J Pers Soc Psychol. 2014; 106, 526–545. 10.1037/a0035663 24564373

[18] Viki GT, Winchester L, Titshall L, Chisango T, Pina A, Russell R. Beyond secondary emotions: The infrahumanization of outgroups using human-related and animal-related words. Soc Cogn. 2006; 24: 753–775.

[19] Haslam N. Dehumanization: An integrative review. Pers Soc Psychol Rev. 2006; 10: 252–264. 10.1207/s15327957pspr1003_4 16859440

[20] Andrighetto L, Baldissarri C, Lattanzio S, Loughnan S, Volpato C. Human-itarian aid? Two forms of dehumanization and willingness to help after natural disasters. Br J Soc Psychol. 2014; 53, 573–584. 10.1111/bjso.12066 24588786

[21] Waytz A, Schroeder J. Overlooking others: Dehumanization by commission and omission. TPM Test Psychom Methodol Appl Psychol. 2014; 21: 251–266.

[22] Waytz A, Schroeder J, Epley N. The lesser minds problem In: Bain PG, Vaes J, Leyens J-P, editors. Humanness and dehumanization. New York: Psychology Press; 2014 pp. 49–67.

[23] Greitemeyer T, McLatchie N. Denying humanness to others: A newly discovered mechanism by which violent video games increase aggressive behavior. Psychol Sci. 2011; 22: 659–665. 10.1177/0956797611403320 21422464

[24] Viki GT, Osgood D, Phillips S. Dehumanization and self-reported proclivity to torture prisoners of war. J Exp Soc Psychol. 2013; 49: 325–328.

[25] Rudman LA, Mescher K. Of animals and objects: Men’s implicit dehumanization of women and likelihood of sexual aggression. Pers Soc Psychol Bull. 2012; 38: 734–746. 10.1177/0146167212436401 22374225

[26] Trifiletti E, Di Bernardo GA, Falvo R, Capozza D. Patients are not fully human: A nurse's coping response to stress. J Appl Soc Psychol. 2014; 44: 768–777.

[27] Vaes J, Muratore M. Defensive dehumanization in the medical practice: A cross-sectional study from a health care worker’s perspective. Br J Soc Psychol. 2013; 52: 180–190. 10.1111/bjso.12008 23013264

[28] Gaunt R. Superordinate categorization as a moderator of mutual infrahumanization. Group Process Intergroup Relat. 2009; 12: 731–746.

[29] Hodson G, Costello K. The human cost of devaluing animals. New Sci. 2012; 216: 34–35.

[30] Hodson G, Kteily N, Hoffarth M. Of filthy pigs and subhuman mongrels: Dehumanization, disgust, and intergroup prejudice. TPM Test Psychom Methodol Appl Psychol. 2014; 21: 267–284.

[31] Albarello F, Rubini M. Reducing dehumanisation outcomes towards Blacks: The role of multiple categorisation and of human identity. Eur J Soc Psychol. 2012; 42: 875–882.

[32] Prati F, Crisp RJ, Meleady R, Rubini M. Humanizing outgroups through multiple categorization: The roles of individuation and threat. Pers Soc Psychol Bull. 2016; 42: 526–539. 10.1177/0146167216636624 26984016 PMC4795148

[33] Pettigrew TF, Tropp LR. A meta-analytic test of intergroup contact theory. J Pers Soc Psychol. 2006; 90: 751–783. 10.1037/0022-3514.90.5.751 16737372

[34] Pettigrew TF, Tropp LR. When groups meet: The dynamics of intergroup contact. London: Psychology Press; 2011.

[35] Capozza D, Falvo R, Favara I, Trifiletti E. The relationship between direct and indirect cross-group friends and outgroup humanization: Emotional and cognitive mediators. TPM Test Psychom Methodol Appl Psychol. 2013; 20: 383–398.

[36] Wright SC, Aron A, McLaughlin-Volpe T, Ropp SA. The extended contact effect: Knowledge of cross-group friendships and prejudice. J Pers Soc Psychol. 1997; 73: 73–90.

[37] Crisp RJ, Turner RN. The imagined contact hypothesis. Adv Exp Soc Psychol. 2012; 46: 125–182.

[38] Kawakami K, Phills CE, Steele JR, Dovidio JF. (Close) distance makes the heart grow fonder: Improving implicit racial attitudes and interracial interactions through approach behaviors. J Pers Soc Psychol. 2007; 92: 957–971. 10.1037/0022-3514.92.6.957 17547482

[39] Woud ML, Maas J, Becker ES, Rinck M. Make the manikin move: Symbolic approach-avoidance responses affect implicit and explicit face evaluations. J Cogn Psychol. 2013; 25: 738–744.

[40] Gawronski B, LeBel EP. Understanding patterns of attitude change: When implicit measures show change, but explicit measures do not. J Exp Soc Psychol. 2008; 44: 1355–1361.

[41] Grumm M, Nestler S, vonCollani G. Changing explicit and implicit attitudes: The case of self-esteem. J Exp Soc Psychol. 2009; 45: 327–335.

[42] Olson MA, Fazio RH. Reducing automatically activated racial prejudice through implicit evaluative conditioning. Pers Soc Psychol Bull. 2006; 32: 421–433. 10.1177/0146167205284004 16513796

[43] De Houwer J, Crombez G, Baeyens F, Hermans D. On the generality of the affective Simon effect. Cogn Emot. 2001; 15: 189–206.

[44] Karpinski A, Hilton JL. Attitudes and the Implicit Association Test. J Pers Soc Psychol. 2001; 81: 774–788. 11708556 10.1037//0022-3514.81.5.774

[45] Krieglmeyer R, Deutsch R. Comparing measures of approach-avoidance behaviour: The manikin task vs. two versions of the joystick task. Cogn Emot. 2010; 24: 810–828.

[46] Chen M, Bargh JA. Consequences of automatic evaluation: Immediate behavioral predispositions to approach or avoid the stimulus. Pers Soc Psychol Bull. 1999; 25: 215–224.

[47] Rinck M, Becker ES. Approach and avoidance in fear of spiders. J Behav Ther Exp Psychiatry. 2007; 38: 105–120. 10.1016/j.jbtep.2006.10.001 17126289

[48] Phills CE, Kawakami K, Tabi E, Nadolny D, Inzlicht M. Mind the gap: Increasing associations between the self and blacks with approach behaviors. J Pers Soc Psychol. 2011; 100: 197–210. 10.1037/a0022159 21299313

[49] Huijding J, Muris P, Lester KJ, Field AP, Joosse G. Training children to approach or avoid novel animals: Effects on self-reported attitudes and fear beliefs and information-seeking behaviors. Behav Res Ther. 2011; 49: 606–613. 10.1016/j.brat.2011.06.005 21763640

[50] Pettigrew TF, Tropp LR. How does intergroup contact reduce prejudice? Meta-analytic tests of three mediators. Eur J Soc Psychol. 2008; 38: 922–934.

[51] Tam T, Hewstone M, Kenworthy J, Cairns E. Intergroup trust in Northern Ireland. Pers Soc Psychol Bull. 2009; 35: 45–59. 10.1177/0146167208325004 19106077

[52] Tropp LR. The role of trust in intergroup contact: Its significance and implications for improving relations between groups In: Wagner U, Tropp LR, Finchilescu G, Tredoux C, editors. Improving intergroup relations: Building on the legacy of Thomas F. Pettigrew. Oxford: Blackwell; 2008 pp. 91–106.

[53] Turner RN, West K, Christie Z. Outgroup trust, intergroup anxiety, and outgroup attitude as mediators of the effect of imagined intergroup contact on intergroup behavioral tendencies. J Appl Soc Psychol, 2013; 43: E196–E205.

[54] Turner RN, Hewstone M, Voci A, Vonofakou C. A test of the extended intergroup contact hypothesis: The mediating role of intergroup anxiety, perceived ingroup and outgroup norms, and inclusion of the outgroup in the self. J Pers Soc Psychol. 2008; 95: 843–860. 10.1037/a0011434 18808263

[55] Vezzali L, Hewstone M, Capozza D, Giovannini D, Wölfer R. Improving intergroup relations with extended and vicarious forms of indirect contact. Eur Rev Soc Psychol. 2014; 25: 314–389.

[56] Crisp RJ, Turner RN. Imagined intergroup contact: Refinements, debates, and clarifications In Hodson G, Hewstone M., editors. Advances in intergroup contact. London: Psychology Press; 2013 pp. 135–151.

[57] Bargh JA, Chartrand TL. The mind in the middle: A practical guide to priming and automaticity research In: Reis HT, Judd CM, editors. Handbook of research methods in social and personality psychology. New York: Cambridge University Press; 2000 pp. 253–285.

[58] Kawakami K, Dovidio JF, Moll J, Hermsen S, Russin A. Just say no (to stereotyping): Effects of training in the negation of stereotypic associations on stereotype activation. J Pers Soc Psychol. 2000; 78: 871–888. 10821195 10.1037//0022-3514.78.5.871

[59] Kawakami K, Dovidio JF, van Kamp S. Kicking the habit: Effects of nonstereotypic association training and correction processes on hiring decisions. J Exp Soc Psychol. 2005; 41: 68–75.

[60] Greenwald AG, McGhee DE, Schwartz JL. Measuring individual differences in implicit cognition: The Implicit Association Test. J Pers Soc Psychol. 1998; 74: 1464–1480. 9654756 10.1037//0022-3514.74.6.1464

[61] Greenwald AG, Nosek BA, Banaji MR. Understanding and using the Implicit Association Test: I. An improved scoring algorithm. J Pers Soc Psychol. 2003; 85: 197–216. 12916565 10.1037/0022-3514.85.2.197

[62] Wiers RW, Rinck M, Kordts R, Houben K, Strack F. Retraining automatic action‐tendencies to approach alcohol in hazardous drinkers. Addiction. 2010; 105: 279–287. 10.1111/j.1360-0443.2009.02775.x 20078486

[63] Wiers RW, Houben K, Fadardi JS, van Beek P, Rhemtulla M, Cox WM. Alcohol cognitive bias modification training for problem drinkers over the web. Addict Behav. 2015; 40: 21–26. 10.1016/j.addbeh.2014.08.010 25218067

[64] Lai CK, Cooley E, Devos T, Xiao YJ, Simon S, Joy-Gaba JA, et al Reducing implicit racial preferences: II. Intervention effectiveness across time. J Exp Soc Psychol. 2016; 145: 1001–1016. 10.1037/xge0000179 27454041

[65] Stephan WG, Stephan CW. Intergroup anxiety. J Soc Issues. 1985; 41: 157–175.

[66] Hayes AF. Introduction to mediation, moderation, and conditional process analysis. New York: Guilford Press; 2013.

[67] Hodson G, Dube B, Choma BL. Can (elaborated) imagined contact interventions reduce prejudice among those higher in intergroup disgust sensitivity (ITG‐DS)? J Appl Soc Psychol. 2015; 45: 123–131.

[68] Meleady R, Seger CR. Imagined contact encourages prosocial behavior towards outgroup members. Group Process Intergroup Relat. 2016; 1–18. Advance online publication.

[69] Pagotto L, Visintin EP, De Iorio G, Voci A. Imagined intergroup contact promotes cooperation through outgroup trust. Group Process Intergroup Relat. 2013; 16: 209–216.

[70] Haslam N, Stratemeyer M. Recent research on dehumanization. Curr Opin Psychol. 2016; 11: 25–29.

[71] Eyssel F, Ribas X. How to be good (or bad): On the fakeability of dehumanization and prejudice against outgroups. Group Process Intergroup Relat. 2012; 15: 804–812.

[72] Lewicki RJ, McAllister DJ, Bies RJ. Trust and distrust: New relationships and realities. Acad Manage Rev. 1998; 23: 438–458.

[73] Swart H, Hewstone M, Christ O, Voci A. Affective mediators of intergroup contact: A three-wave longitudinal study in South Africa. J Pers Soc Psychol. 2011; 101: 1221–1238. 10.1037/a0024450 21728450

[74] Al Ramiah A, Hewstone M, Voci A, Cairns E, Hughes J. It's never too late for ‘us’ to meet ‘them’: Prior intergroup friendships moderate the impact of later intergroup friendships in educational settings. Br J Educ Psychol. 2013; 83: 57–75. 10.1111/j.2044-8279.2011.02054.x 23369175

[75] Davies K, Wright SC, Aron A, Comeau J. Intergroup contact through friendship: Intimacy and norms In: Hodson G, Hewstone M, editors. Advances in intergroup contact. London: Psychology Press; 2013 pp. 200–230.

