# Supplementary material for: Intergroup Contact and Outgroup Humanization: Is the Causal Relationship Uni- or Bidirectional?
Source: PLoS One. 2017 Jan 24;12(1):e0170554. doi: 10.1371/journal.pone.0170554 (PMC5261613; doi:10.1371/journal.pone.0170554)
Supplement: S1 Appendix — (DOCX) [file pone.0170554.s001.docx]

**S1 Appendix. Unconditioned stimuli used in the procedure of subliminal conditioning, Study 1.**

*Humanization condition.* Uniquely human traits: intellectual abilities [raziocinio], morality [moralità], rationality [razionalità], reasoning [ragione]. Uniquely human emotions: admiration [ammirazione], optimism [ottimismo], pride [orgoglio]; regret [rimpianto], remorse [rimorso], shame [vergogna]. Human concepts: bachelor [scapolo], citizen [cittadino], human [umano], humans [umani], young boy [fanciullo].

*Dehumanizing condition.* Non-uniquely human traits: drive [pulsione], impetus [impeto], impulsiveness [impulsività], instinct [istinto]. Non-uniquely human emotions: excitement [eccitazione], pleasure [piacere], surprise [sorpresa]; pain [dolore], rage [rabbia], sadness [tristezza]. Animal concepts: animal [animale], animals [animali], cub [cucciolo], fauna [fauna], specimen [esemplare].

Note: The Italian version of the stimuli used in the experiment are shown in brackets.
